# Report of a Rare Case of Acute Abdominal Pain Post-partum: Spontaneous Ureteral Rupture

**DOI:** 10.7759/cureus.76531

**Published:** 2024-12-28

**Authors:** Parkha Khan, Doaa A Ibrahim, Vinita Meena

**Affiliations:** 1 General Internal Medicine, Leeds Teaching Hospitals NHS Foundation Trust, Leeds, GBR; 2 Surgery, Al Tahrir General Hospital, Cairo, EGY; 3 Radiology, West Suffolk NHS Foundation Trust, Bury St Edmunds, GBR

**Keywords:** abdominal pain, ct urogram, pregnancy, ureteric rupture, urinoma

## Abstract

Spontaneous ureteral rupture is a rare cause of acute abdominal pain, particularly unusual during pregnancy or the post-partum period. While pregnancy-related changes like ureteral compression and dilation may play a role, no definitive mechanisms have been established. Clinicians should suspect ureteric injury in post-partum patients with free pelvic fluid. Diagnosis relies on contrast-enhanced CT and cystoureteroscopy, with ureteral stenting being an effective management strategy. Our case is a 32-year-old primigravida (G1 P1) woman who presented with acute abdominal pain shortly after forceps-assisted vaginal delivery. Physical examination revealed right iliac fossa rebound tenderness. Laboratory tests showed elevated inflammatory markers, increased creatinine levels, and reduced estimated glomerular filtration rate (eGFR), indicating stage 2 acute kidney injury. Contrast-enhanced CT of the abdomen and pelvis demonstrated free-fluid collection with air foci and contrast extravasation in the vicinity of the distal segment of the right ureter, confirming ureteric rupture. The patient was treated with intravenous antibiotics and intravenous fluids. A multidisciplinary team meeting decided on definitive management through a nephrostomy procedure, which was unsuccessful. Subsequently, surgical repair was performed, involving a ureteral stent and a bladder patch placement to facilitate the healing of the ureteral tear. The ureteric stent was removed six weeks later via cystoscopy after a retrograde pyelogram confirmed complete healing of the tear. In conclusion, spontaneous ureteric rupture is a rare and underreported complication of vaginal delivery and is more common to occur in the renal fornices and upper ureter. CT urogram serves as the gold standard for diagnosis. Severe abdominal pain in the postpartum period can point to its occurrence. Treatment is usually by a nephrostomy or a ureteric stent with or without surgical reconstruction of the ureter.

## Introduction

Spontaneous ureteric rupture is a very rare and serious event that can happen in the post-partum period. It can lead to several dangerous complications if not properly recognized and managed, including urinoma, retroperitoneal abscess, and systemic sepsis [1].

Commonly reported causes of ureteric rupture include calculi, malignancy, trauma, and retroperitoneal fibrosis [2]. Only a few reported cases of spontaneous urinary tract rupture are in the literature. The rupture commonly occurs in the renal fornices and upper ureter [3]. Such pathology is even rarer in pregnant women without preexisting urinary tract pathology [4].

This article reports a rare case of spontaneous ureteric rupture after normal vaginal delivery of a primigravida lady without any previously known urological disorders and the challenging management of this case.

## Case presentation

A 32-year-old lady primigravida (G1 P1) was admitted at 38 weeks as part of the management of her gestational diabetes. The baby weighed more than the 90^th^ centile on her antenatal scans and was large for gestational age (LGA). Her physical examination was unremarkable at the time of presentation. The patient’s admission laboratory results are indicated in Table 1.

**Table 1:** Laboratory results on admission

Parameter	Result	Normal value
Hemoglobin (Hb) (g/L)	120	115-165
White blood cells (x10^9^/L)	11	3.6-11
Creatinine (µmol/L)	54	45-84
C-reactive protein (CRP) (mg/L)	4	0-5

After six hours of admission, the patient had a spontaneous rupture of membranes. The treating team planned to allow the natural progression of labor. At 12 hours, the labor did not progress successfully with inadequate cervical dilatation. Therefore, labor was induced with oxytocin infusion, reaching up to 0.02 units/minute. She entered a prolonged second stage of labor and hence decided to undergo forceps-assisted delivery. A right mediolateral episiotomy was made with an Episcissor, and Rhodes forceps were used for assisted delivery. The lady delivered a healthy baby weighing 3290 grams without associated complications. She passed clear urine within four hours after delivery, and the indwelling urinary catheter was removed.

**Figure 1:**
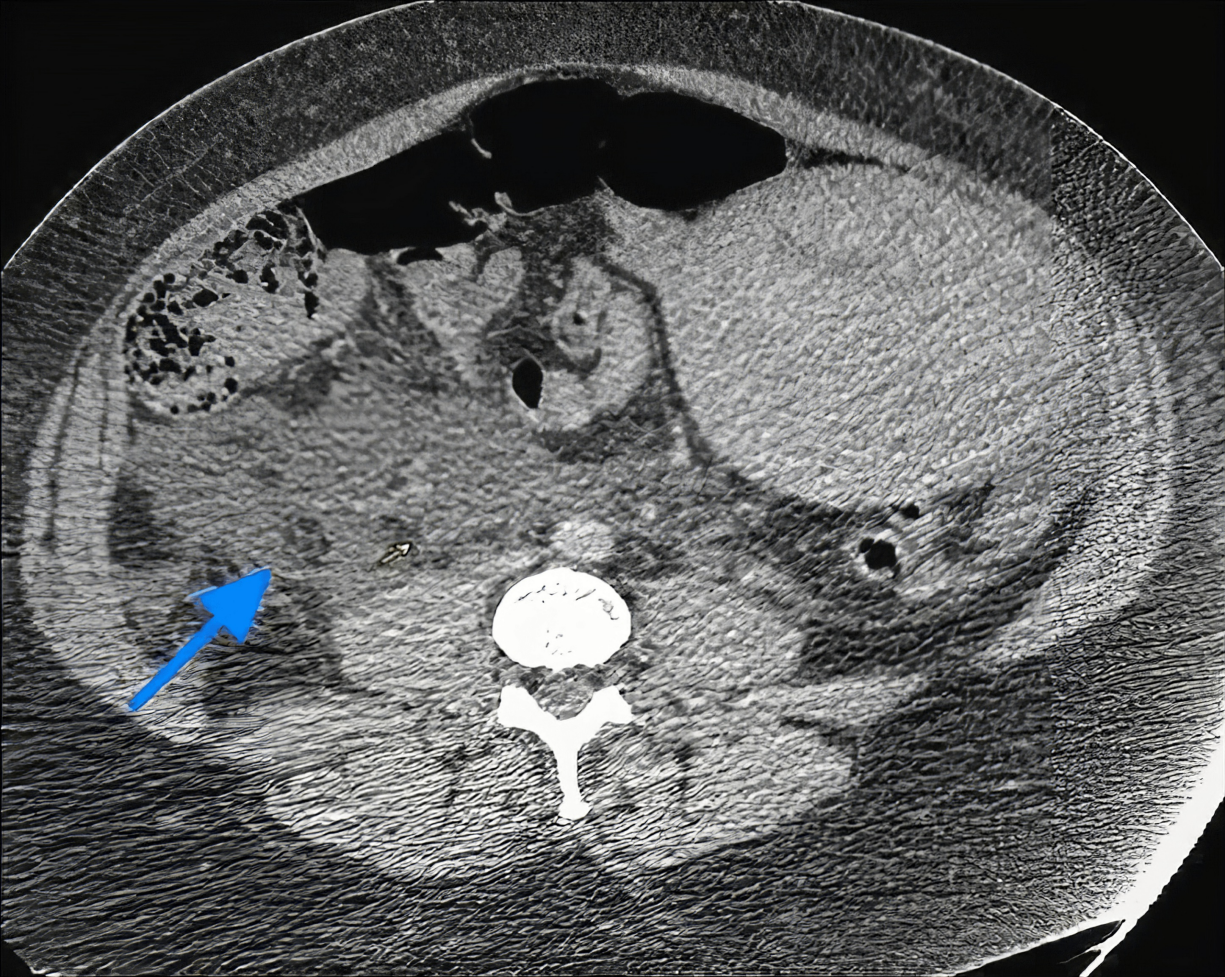
CT post-contrast portal venous phase axial image showing moderate free-fluid collection with air foci adjacent to the lower segment of the right ureter (arrow). An enlarged post-partum uterus can be noted on the left side of the lower abdomen.

**Figure 2:**
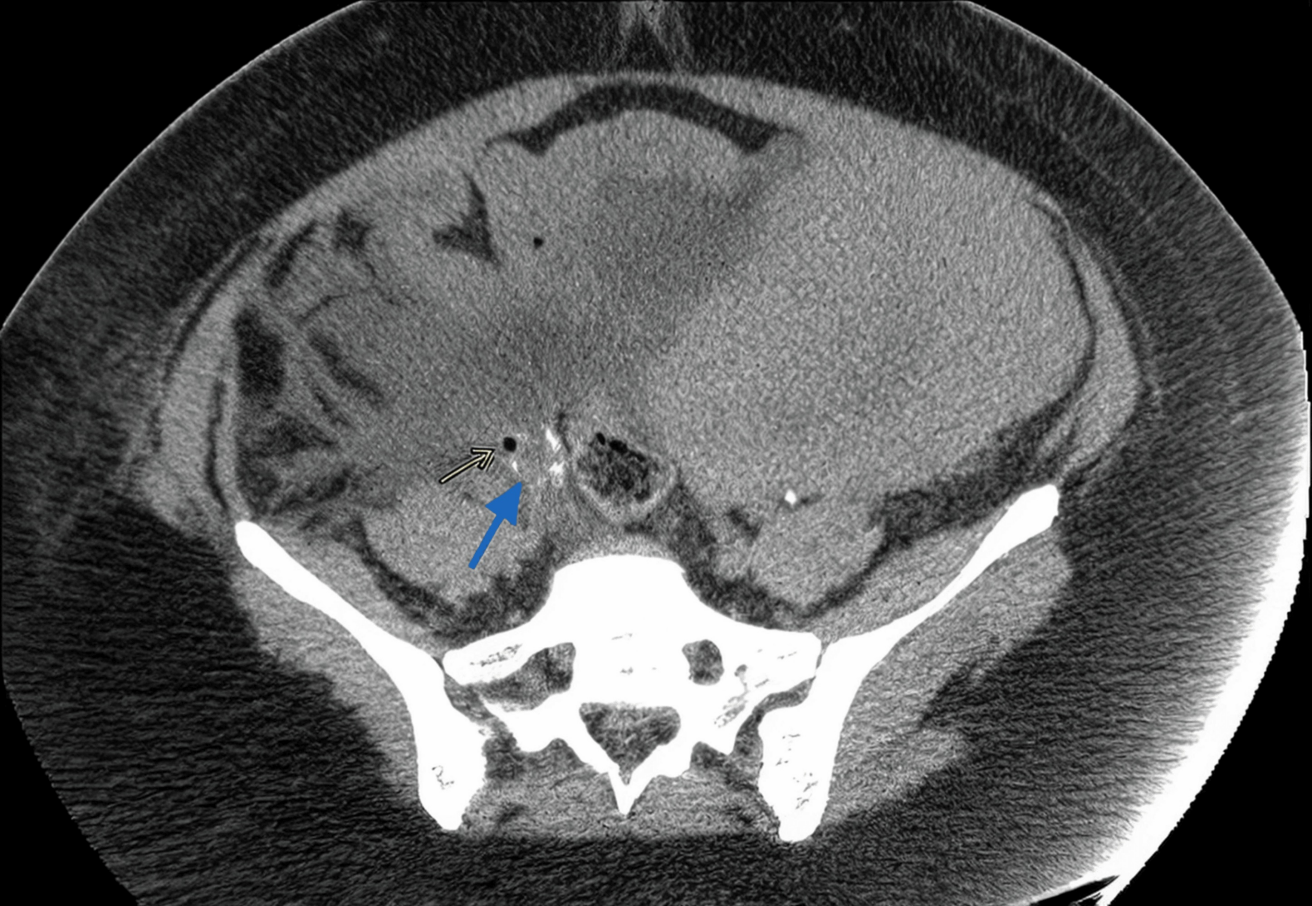
CT post-contrast delayed phase axial image: confirming the extravasation of contrast from the distal segment of the right ureter just distal to the right iliac vessels bifurcation (arrow). Air foci near the lumen of the right ureter confirm the site of the leak.

**Figure 3:**
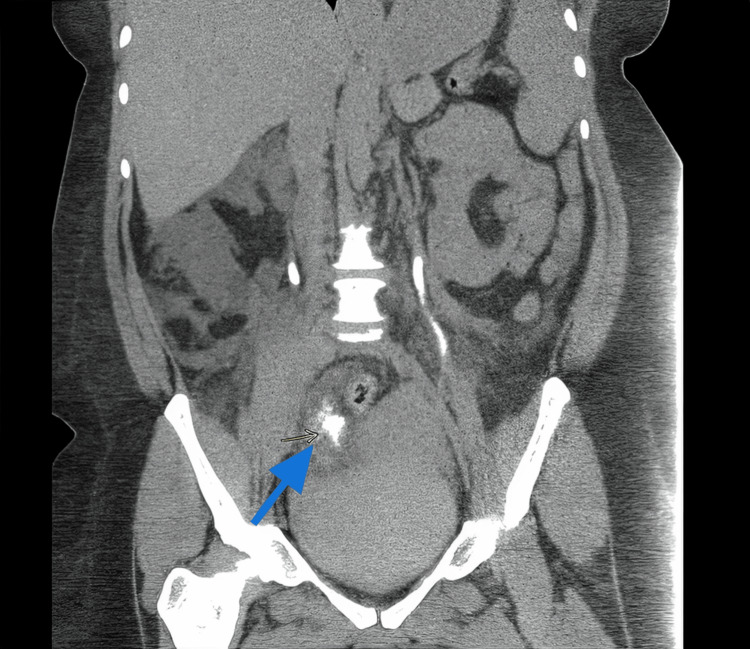
CT post-contrast delayed phase coronal image: showing extravasation of contrast near the distal right ureteric course (arrow). The left ureter appears normal in course and caliber.

On day one of delivery, the patient complained of worsening, dull, right-sided flank pain requiring opioid analgesia. On examination, she had rebound tenderness in the right iliac fossa. She became febrile (temperature was 38.6 °C), tachycardic (heart rate was 120 beats per minute), and hypotensive ( blood pressure was 88/60 mmHg). Repeat blood tests showed a Hb drop, with a rise in the white cell count, creatinine (indicating a stage 2 acute kidney injury), and CRP (Table 2). An initial differential diagnosis included appendicitis, uterine rupture, puerperal sepsis, and kidney calculi. She underwent a CT scan of the abdomen and pelvis with IV contrast, with image acquisition at 70 seconds showing right flank and pelvis fluid attenuation collection, suggesting a urinoma with air foci near the right ureter lumen (Figure 1). Then, the delayed phase was consecutively acquired after 10 minutes of initial intravenous contrast injection that showed active contrast extravasation along the course of the distal right ureter, suggesting urine extravasation (Figures 2, 3). The patient was started on IV crystalloids, IV ceftriaxone, IV metronidazole, and a single dose of IV gentamicin.

**Table 2:** Changes in laboratory results on day 1 after delivery

Parameter	Result on day 1	Result on admission	Normal value
Hemoglobin (g/L)	99	120	115-165
White blood cells (x10^9^/L)	13.7	11	3.6-11
Creatinine (µmol/L)	156	54	45-84
C-reactive protein (CRP) (mg/L)	104	4	0-5

The case was discussed in a multidisciplinary team meeting, including an interventional radiologist, a urologist, and a gynecologist. The team agreed on going for surgical nephrostomy. The nephrostomy procedure was unsuccessful owing to the difficulty of inserting the nephrostomy tube into a non-dilated right ureter. Consequently, the right ureter was repaired using a stent and a bladder patch placement on the ureter. The patient was discharged the next day in a stable condition after improvement of her renal functions and inflammatory markers.

A few days after the repair, she presented to the emergency department with a recurrence of right flank pain. The urology team assessed her, and a repeat contrast CT scan of the abdomen and pelvis showed fluid collection around the repaired ureter. The interventional radiology team drained the collection, which significantly improved the pain. At six weeks, she underwent a cystoscopy with a retrograde pyelogram that confirmed the absence of a ureteric contrast leak. The stent was subsequently removed without complications.

## Discussion

We reported an uncommon case of a spontaneous ureteric rupture in a primigravida woman without preceding urinary tract disease. The rupture was complicated by the formation of a urinoma and was repaired using a patch and a ureteric stent. This case report highlights a rare cause of acute abdominal pain in post-partum ladies that, if not managed in a timely manner, can lead to potentially life-threatening complications.

Spontaneous rupture of the ureter is a rare cause of acute abdominal pain. It implies ureteral damage without external trauma, calculi, or kidney disease [4]. Rupture of the renal collecting system may happen when the intraluminal pressure exceeds the critical threshold of 70-75 mmHg [5]. Such pressure rise can be observed in physiological pregnancy-induced hydronephrosis due to hormonal and mechanical factors [6].

The differential diagnosis of post-partum ureteral rupture is broad and includes urolithiasis, cholecystitis, appendicitis, puerperal infection, and uterine rupture [4]. The gold standard investigation is a CT scan of the abdomen and pelvis with IV contrast, demonstrating contrast extravasation from the ureter [7]. Initial treatment involves medical resuscitation with IV fluids and IV antibiotics, as well as close monitoring of the patient’s observations. Definitive treatment includes a ureteric stent insertion or the performance of a nephrostomy [8,9]. Our case needed a bladder patch reconstruction of the ureter and the ureteric stent insertion.

The location of the ureteral rupture in our case is unique, involving the lower segment of the ureter. On the other hand, the renal fornices and the upper ureter are reported to be the commonest sites of rupture [3]. Such uniqueness led to our proposition that the possible cause of the rupture could be the rapid changes in abdominal pressures during the induction of delivery in the presence of high intraluminal pressures secondary to the physiological hydronephrosis of pregnancy [10]. Some external avulsion forces from the instrumentation to assist delivery may have contributed to the rupture.

## Conclusions

Ureteric rupture should be considered in the differential diagnosis of acute abdominal pain in the post-partum period. Missing the diagnosis and not managing it promptly can lead to grave consequences, including the formation of a urinoma or abscess and sepsis. CT urogram is the gold standard diagnostic tool to confirm the condition. Treatment is through a nephrostomy or ureteric stent with or without ureteric construction.
